# Dorsal displacement of the facial nerve in vestibular schwannoma surgery

**DOI:** 10.3171/2021.7.FOCVID2182

**Published:** 2021-10-01

**Authors:** Gustavo S. Jung, Joel Fernando Sanabria Duarte, Afonso H. de Aragão, Ronaldo Pereira Vosgerau, Ricardo Ramina

**Affiliations:** 1 Department of Neurological Surgery, Skull Base Division;; 2 Department of Neurological Surgery, Post-Graduate Program; and; 3 Department of Radiology, Neuroradiology Division, Neurological Institute of Curitiba, Paraná, Brazil

**Keywords:** acoustic neuroma, facial nerve, facial palsy, vestibular schwannoma

## Abstract

The course of the facial nerve (FN) has been extensively investigated in patients with vestibular schwannomas (VSs). FN running dorsally to the tumor capsule accounts for less than 3% of the cases. Diffusion tensor imaging (DTI)–based fiber tracking helps to preoperatively identify the FN. During surgery, a higher risk of injury is associated with the dorsal location of the FN. The authors demonstrate the nuances and tricks to identify and preserve a dorsal displaced FN during resection of a large VS, T3b according to the Hannover classification, through the retrosigmoid-transmeatal approach.

The video can be found here: https://stream.cadmore.media/r10.3171/2021.7.FOCVID2182

## Transcript

This is Dr. Gustavo Jung and I’ll demonstrate the microsurgical resection of a vestibular schwannoma with the facial nerve dorsally displaced and the technique to identify the course of the facial nerve during surgery.

### 0:35 Patient History

This 57-year-old male discovered a large vestibular schwannoma during a diagnostic workup for right-side tinnitus. It was classified as a T3b vestibular schwannoma according to the Hannover classification and stage III according to Koos classification. The patient had an AAOHNS hearing class B and a House-Brackmann grade I facial nerve function.

### 1:03 Preoperative Imaging

The T1 postgadolinium MRI demonstrated a CP angle mass compressing the brainstem with extension to the most lateral part of the internal auditory canal. The tumor was predominantly solid, and a CSF cleft sign was not visible between the tumor and the brainstem. The CISS MRI did not raise any suspicion on the facial nerve’s position and DTI-based fiber tracking was not acquired in this case.

### 1:36 Patient Positioning, Skin Incision and Craniotomy

The patient was positioned supine with the head turned approximately 60° to the left side. The right shoulder was elevated with a cushion to facilitate head turning. Facial nerve monitoring and brainstem auditory evoked response were used. A straight skin incision 4 cm behind the pinna is designed, and a retrosigmoid craniotomy is elevated in a standard fashion.

### 2:06 Dural Incision and Arachnoid Dissection

The dura is incised in C-shape fashion and a perpendicular cut is made toward the sigmoid-transverse junction to increase the view angle of the petrous surface. The dura is elevated with 4-0 Prolene sutures.

### 2:22 Tumor Exposure

The inferior surface of the cerebellum is gently retracted, and the cerebellum-medullary cistern is opened to brain relaxation. The microscope is turned toward the tentorial surface of the cerebellum inspecting for cerebellar bridge veins and Dandy’s vein. A small bridge vein is coagulated around the jugular foramen and cut. The arachnoid membrane is adherent to the dorsal surface of the tumor. To avoid injury to the nerves or the tumor capsule, the arachnoid is released from the lower cranial nerves; at this place it is not tightly adherent to the tumor capsule. The arachnoid layer becomes visible at the dorsal surface of the VS. A nerve structure is visible on the dorsal surface of the tumor and EMG activity of the facial nerve is recorded. A further dissection of the arachnoid around the lower cranial nerves is necessary to expose the brainstem. A progressive 1.0- and 2.0-mA stimulus is used and only mild response is observed. The brainstem is inspected, and the facial nerve is visualized running dorsally to the tumor capsule. The inferior vestibular nerve and the proximal portion of the facial nerve are identified. Some mild EMG response is also evoked during the stimulation of the upper pole of the tumor, making unpredictable the precise course of the facial nerve.

### 4:10 Internal Auditory Canal Drilling

To better delineate the trajectory of the facial nerve, the distal portion of the FN is exposed inside the IAC. A dural flap is harvested and kept attached over the jugular foramen. We start drilling the IAC with a large cutting burr, creating a safety cavity to prevent the drill slide. Smaller cutting burrs are used until an eggshell bone is left over the IAC. Diamond drills are used to expose the dura inside the IAC and enlarge the bone cavity sufficiently to access the lateral portion of the tumor.

### 4:54 Removal of Intrameatal Tumor and Facial Nerve Identification

The dura inside the IAC is incised with microscissors, and the inferior vestibular nerve is dissected away from the tumor. The tumor within the IAC is dissected with a blunt hook dissector and removed. The facial nerve is identified and stimulation on the upper and lower portions of the cisternal-meatal region identify the facial nerve running dorsal inferiorly.

### 5:22 Removal of the Cisternal Tumor

After identifying the facial nerve running dorsal inferiorly, intracapsular tumor debulking is done and the attention is kept to the upper pole of the tumor. The arachnoid is first dissected in the upper pole of the tumor coming from the meatus up to the facial nerve at the brainstem. The inferior portion of the tumor is dissected from lateral to medial, and additional debulking is done. A decrease in the amplitude of waves III and V in the AED is recorded, and papaverine solution is instilled until waves recovery. The cochlear nerve is identified anterior to the inferior vestibular nerve and the facial nerve.

Bimanual dissection with a tumor forceps in nondominant hand and a blunt hook dissector in the dominant hand, the facial nerve is dissected from the tumor capsule all the way to the IAC. Preservation of facial nerve function is confirmed with proximal and distal electric stimulation with 1.0 mA.

### 6:41 Internal Auditory Canal Reconstruction

The vascularized dural flap is rotated into the IAC covering the nerves. A 0.5-mm hook dissector is used to inspect the walls of IAC for the presence of opened mastoid air cells. These cells are occluded with small pieces of muscle, and the IAC is completely occluded with a larger piece of muscle, Surgicel, and fibrin glue.

### 7:07 Dural Closure

The dura is closed in watertight fashion with 4-0 Prolene sutures, and the mastoid air cells are closed with bone wax. The bone flap is repositioned and fixed with titanium plates.

### 7:21 Postoperative Course

Postoperative MRI did not show any residual lesion, and the facial nerve is well visualized in the CISS MRI. The patient remained 1 day in the ICU and was discharged home in the postoperative day 4. The patient remained stable with a House-Brackmann grade III facial palsy that recovered to grade I 4 months after surgery with physiotherapy. Hearing decreased to AAOHNS class C.

### 7:52 Conclusions and References

In summary, vestibular schwannomas are challenging lesions regardless the position of the facial nerve. Dorsal displacement of the facial nerve is rare and accounts for less than 3% of the cases.^1–3^ DTI-based fiber tracking can help to the identify the facial nerve course preoperatively,^4,5^ but its accuracy is intermediate when the nerve is running dorsally.^6^ Starting arachnoid dissection at the lower pole of the tumor (around the jugular foramen—where it is looser) helps to release the arachnoid in the dorsal surface of the tumor. This strategy associated with the neuromonitoring may avoid injury to the tumor capsule and dorsally displaced nerves. In those cases, our strategy is to first identity the nerve at brainstem and then open the IAC to dissect the facial nerve within the IAC. The nerve is usually very adherent to the tumor capsule at the meatus entrance. The electric stimulation of this cisternal-meatal transition of facial nerve will help in its identification and dissection. Thank you.

## Disclosures

The authors report no conflict of interest concerning the materials or methods used in this study or the findings specified in this publication.

## Author Contributions

Primary surgeon: Ramina. Assistant surgeon: Jung. Editing and drafting the video and abstract: Jung, Duarte, de Aragão. Critically revising the work: Jung, Duarte, de Aragão, Ramina. Reviewed submitted version of the work: Jung, Duarte, de Aragão. Approved the final version of the work on behalf of all authors: Jung. Supervision: Ramina. Diagnostic imaging consultant: Vosgerau.
